# Activation of the Nrf2-ARE pathway by the *Alternaria alternata* mycotoxins altertoxin I and II

**DOI:** 10.1007/s00204-016-1726-7

**Published:** 2016-05-13

**Authors:** Katharina Jarolim, Giorgia Del Favero, Gudrun Pahlke, Victoria Dostal, Kristin Zimmermann, Elke Heiss, Doris Ellmer, Timo D. Stark, Thomas Hofmann, Doris Marko

**Affiliations:** 1 Department of Food Chemistry and Toxicology, Faculty of Chemistry, University of Vienna, Währinger Straße 38, 1090 Vienna, Austria; 2 Department of Pharmacognosy, Faculty of Life Sciences, University of Vienna, Althanstraße 14, 1090 Vienna, Austria; 3 Chair of Food Chemistry and Molecular Sensory Science, TU München, Lise-Meitner-Straße 34, 85354 Freising, Germany

**Keywords:** *Alternaria alternata*, Altertoxin I (ATX I), Altertoxin II (ATX II), Nrf2, Glutathione, Confocal/SIM microscopy

## Abstract

The mycotoxins altertoxin I and II (ATX I and II) are secondary metabolites produced by *Alternaria alternata* fungi and may occur as food and feed contaminants, especially after long storage periods. Although the toxic potential of altertoxins has been previously investigated, little is known about the pathways that play a role in their intracellular metabolism. In order to identify potential targets of ATX I and ATX II, the two toxins were tested for interaction with the nuclear factor erythroid-derived 2-like 2/antioxidant response element (Nrf2/ARE) pathway in mammalian cells. This pathway can be activated by various stressors resulting in the expression of enzymes important for metabolism and detoxification. In the present study, only ATX II triggered a concentration-dependent increase in Nrf2-ARE-dependent luciferase expression. Consistently, confocal microscopy revealed an ATX II-induced increase in Nrf2 signal in HT29 intestinal cells. In agreement with these data, ATX II induced the transcription of γ-glutamate cysteine ligase, the key enzyme in catalyzing GSH synthesis of the cells and which is regulated by Nrf2. Further investigations demonstrated that ATX II induced a concentration-dependent depletion of the cellular GSH levels after short incubation time (3 h) and an increase after longer incubation time (24 h). In conclusion, it was demonstrated that ATX II can interact at several levels of the Nrf2-ARE pathway in mammalian cells and that ATX I does not share the same mechanism of action.

**Electronic supplementary material:**

The online version of this article (doi:10.1007/s00204-016-1726-7) contains supplementary material, which is available to authorized users.

## Introduction

**Fig. 1:**
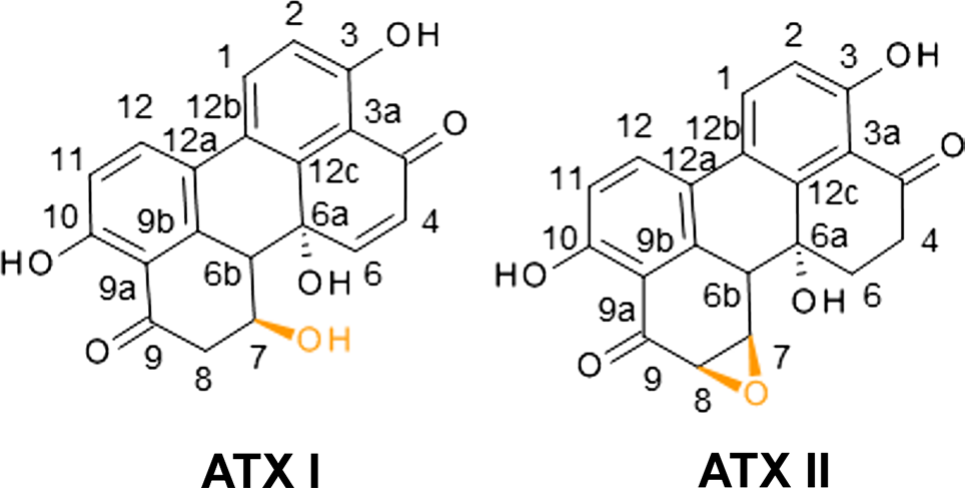
Toxins structures. Chemical structures of the two perylene quinone-type *Alternaria* mycotoxins ATX I and ATX II

The filamentous fungi of the genus *Alternaria* comprise more than 100 species; among these, *Alternaria alternata* is the most common one (Rotem 1994). *A. alternata* is a saprophytic field fungus which is abundant in the atmosphere, in soil and in seeds and has been reported to grow on a wide variety of food and feed items, including fruits like strawberries, blueberries, grapes and citrus fruits (Tournas and Katsoudas 2005), as well as tomatoes (Andersen and Frisvad 2004) and wheat (Müller and Korn 2013). *A. alternata* is known to produce a large number of secondary metabolites and many of these are generally recognized as mycotoxins. For this reason, *A. alternata* might represent a potential health risk for humans and animals, but, in spite of that, no regulatory limits have been introduced so far to define its presence in commercialized food and feed. In this respect, the European Food Safety Authority (EFSA) pointed out the need for further investigations to improve the knowledge about the toxicological profile of mycotoxins formed by *A. alternata* (EFSA 2011). These toxins can be grouped according to their chemical structure, such as the terpenoids, pyranones or quinones (Lou et al. 2013). Among these, the class of the perylene quinone-type mycotoxins comprises ATX I and ATX II (Fig. 1). These two compounds seem to have high potential to pose a major threat for human health due to their mutagenic and genotoxic properties (Schrader et al. 2001; Schwarz et al. 2012b; Stack and Prival 1986). Structurally, the two molecules differ only by means of the functional group in positions 7 and 8, where ATX I carries a hydroxyl group and ATX II an epoxide moiety. Even though minor, this structural difference seems to account for the diverse toxicological characteristics of the two compounds. In fact, both molecules have been described as mutagenic in *S. typhimurium* strains TA98, TA100, TA1537, TA102 and TA104, yet ATX I was reported to be less potent than ATX II (Schrader et al. 2001; Stack and Prival 1986). Similarly, this trend was confirmed for several other properties, such as: mutagenicity in V79 cells (Fleck et al. 2012; Schrader et al. 2006), ability to induce DNA strand breaks and formamidopyrimidine DNA glycosylase-sensitive sites in human cancer cell lines (Fleck et al. 2014; Schwarz et al. 2012b) or cytotoxicity in mammalian cell cultures (Boutin et al. 1989; Tiessen et al. 2013b). Although the toxicological impact of altertoxins has been investigated previously, studies dealing with the metabolic pathways of perylene quinone-type mycotoxins have been limited to the observations that ATX II can be reduced to ATX I by the colorectal adenocarcinoma cell line Caco2 and that ATX II can form adducts with GSH in a cell-free system (Fleck et al. 2014). Thus, many questions remain to be addressed in particular regarding the potential of ATX I and ATX II to induce and/or interact with phase II metabolic pathways. In this context, as a first step, we investigated whether altertoxins interact with the Nrf2-ARE signaling pathway. Nrf2-ARE pathway is a major route triggering the expression of a broad spectrum of phase II enzymes (Itoh et al. 1997). The transcription factor Nrf2 recognizes and binds to the 5′-flanking region of the ARE in the promoter region of target genes which encode for antioxidant proteins and enzymes (Rushmore et al. 1991). Under basal conditions, Nrf2 is negatively regulated by Kelch-like ECH-associated protein 1 (Keap1), which mediates the degradation of the transcription factor via its function as adaptor for the CRL3 class of cullin-RING-ligase E3 (Kobayashi et al. 2004). In oxidative stress conditions, Nrf2 is released from its repressor Keap1 and translocates from the cytosol to the nuclear compartment where it acts as a transcription factor (canonical activation pathway; Nguyen et al. 2009). As a result, Nrf2 plays a vital role in launching cellular response against stressors and inducing important defense systems like glutathione (GSH). Taking this as starting point, the aim of the present study was to investigate whether altertoxins ATX I and II could have an impact on several key steps of the Nrf2-ARE signaling pathway. The effect of the two mycotoxins was investigated for their potential to induce oxidative stress and cytotoxicity, for their effect on the NRF2/Keap1 homeostasis and, ultimately, on the direct effects on the γGCL and cellular GSH.

## Results

### Identification of ATX I and II from *A. alternata* infested rice

MS measurements of ATX I identified the toxin with an *m/z* in the (−) HRESIMS of 351.0868 [M–H]^−^ (calculated for C_20_H_15_O_6_, −0.3 ppm). The NMR data (^1^H NMR and ^13^C NMR) are in line with Hradil et al. (1989) and Stack et al. (1986) (Online Resource). ATX II was identified with an m/z of 349.0731 [M–H]^−^ in the (−) qTOF-MS (calculated for C_20_H_14_O_6_, −3,9 ppm). ATX I and ATX II were isolated from the inoculated rice cultures with purities of >96 and >97 %, respectively.

### Effect of ATXs on the Nrf2-ARE pathway activation in the luciferase reporter gene assay

**Fig. 2:**
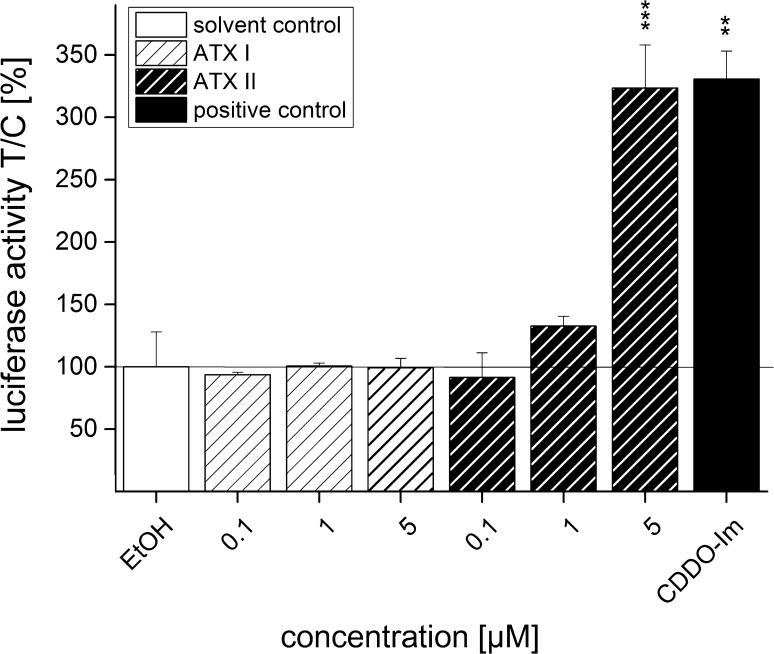
Induction of ARE-dependent luciferase expression after 20 h of incubation with ATX I and ATX II in CHO cells. ARE-driven luciferase activity after 20 h of incubation with ATX I (*striped bars*, *white background*), ATX II (*striped bars*, *black background*) and 0.1 µM CDDO-Im (*positive control*, *black bar*) in serum-containing medium. Luciferase activity data are expressed as mean values ±SEM of at least three independent experiments, calculated to respective GFP luminescence, normalized to solvent control (EtOH). Significance levels refer to a comparison to solvent control (**p < 0.01, ***p < 0.001; *n* = 3 independent experiments performed in triplicate)

In order to investigate whether incubation with ATX I and ATX II can lead to Nrf2-activation, an ARE-dependent luciferase reporter gene assay was performed. In this system, binding of Nrf2 to the ARE promoter region leads to the expression of luciferase, whose activity can be measured by chemiluminescence. After 20 h of incubation, ATX I did not elicit Nrf2 activation in the reporter cells in any of the tested concentrations (Fig. 2). On the contrary, when cells were incubated with ATX II, the Nrf2 activity increased in a concentration-related manner. Incubation of the cells with 5 μM ATX II induced luciferase activity, indicative for activation of the Nrf2-ARE pathway, significantly (323 ± 35 % increase) in comparison with the solvent control (0.1 % EtOH). The luciferase signal measured in cells incubated with 5 μM ATX II reached values comparable to those of the positive control CDDO-Im (331 ± 23 %; Fig. 2). The measurement of the fluorescence of eGFP, which was used as reference for cellular density, showed a slight, though not significant, decrease in cells incubated with 5 µM ATX II (79 ± 19 %) in comparison with the of solvent control (Supplementary material, Fig S1).

### Effect of ATXs on Nrf2 and Keap1 immunolocalization in HT29 cells

**Fig. 3:**
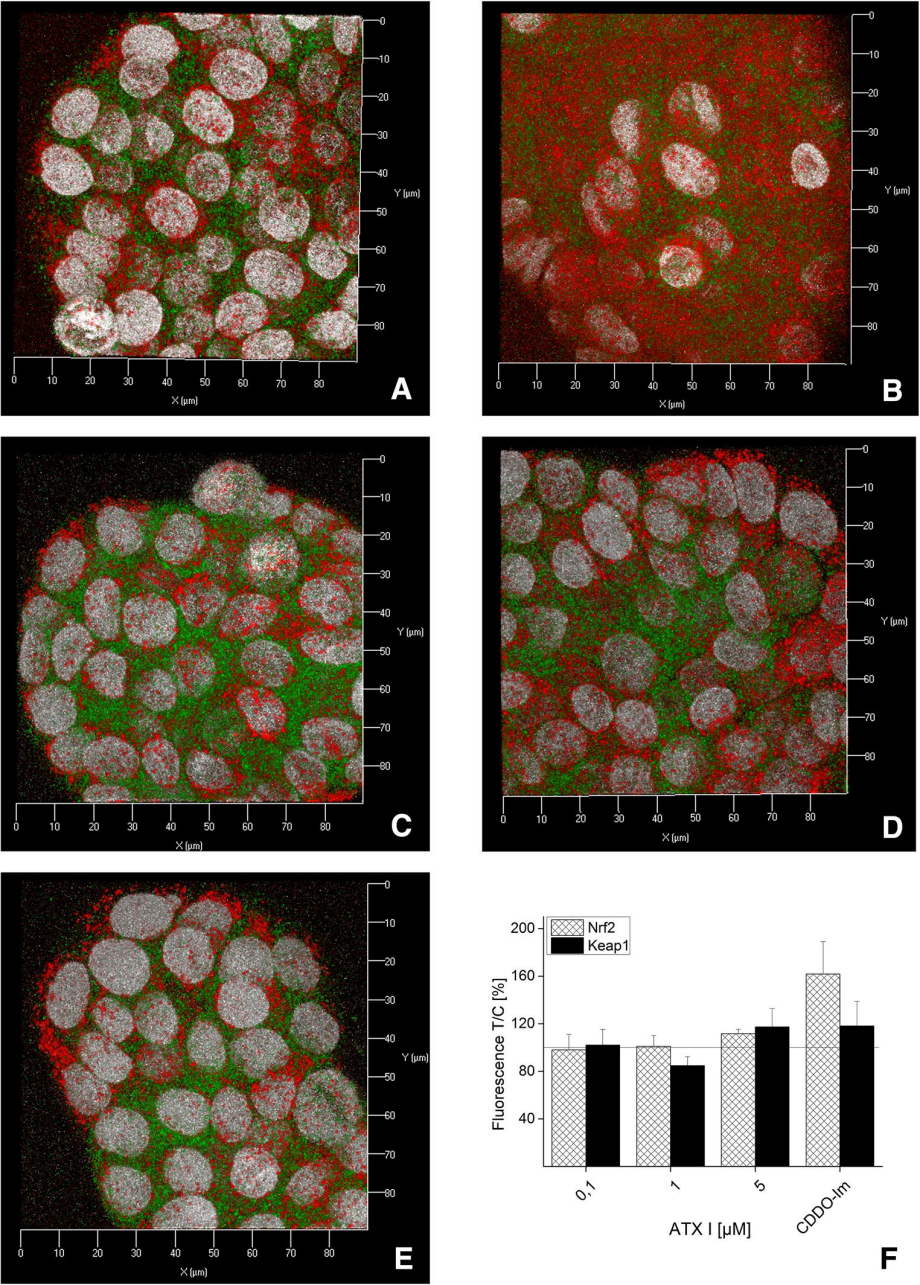
Immunofluorescence localization of Nrf2 and Keap1 after 1 h of incubation with ATX I in HT29 cells. Representative images of immunolocalization of Nrf2 (*red*), Keap-1 (*green*) and lamin B (*gray*) after incubation with EtOH (solvent control, **a**), 0.5 µM CDDO-Im (**b**), ATX I 0.1 µM (**c**), 1 µM (**d**), 5 µM (**e**). *Axes* *x* 0–90 µm, *y* 0–90 µm. **f** Quantification of Nrf2 (*patterned bars*) and Keap1 (*black bars*) signals in cells incubated with ATX I (0.1, 1 and 5 µM) and CDDO-Im (0.5 µM). Mean values of fluorescence in the optical fields are expressed as percentage of solvent controls ±SEM (*n* = 3–6 independent experiments) (color figure online)

**Fig. 4:**
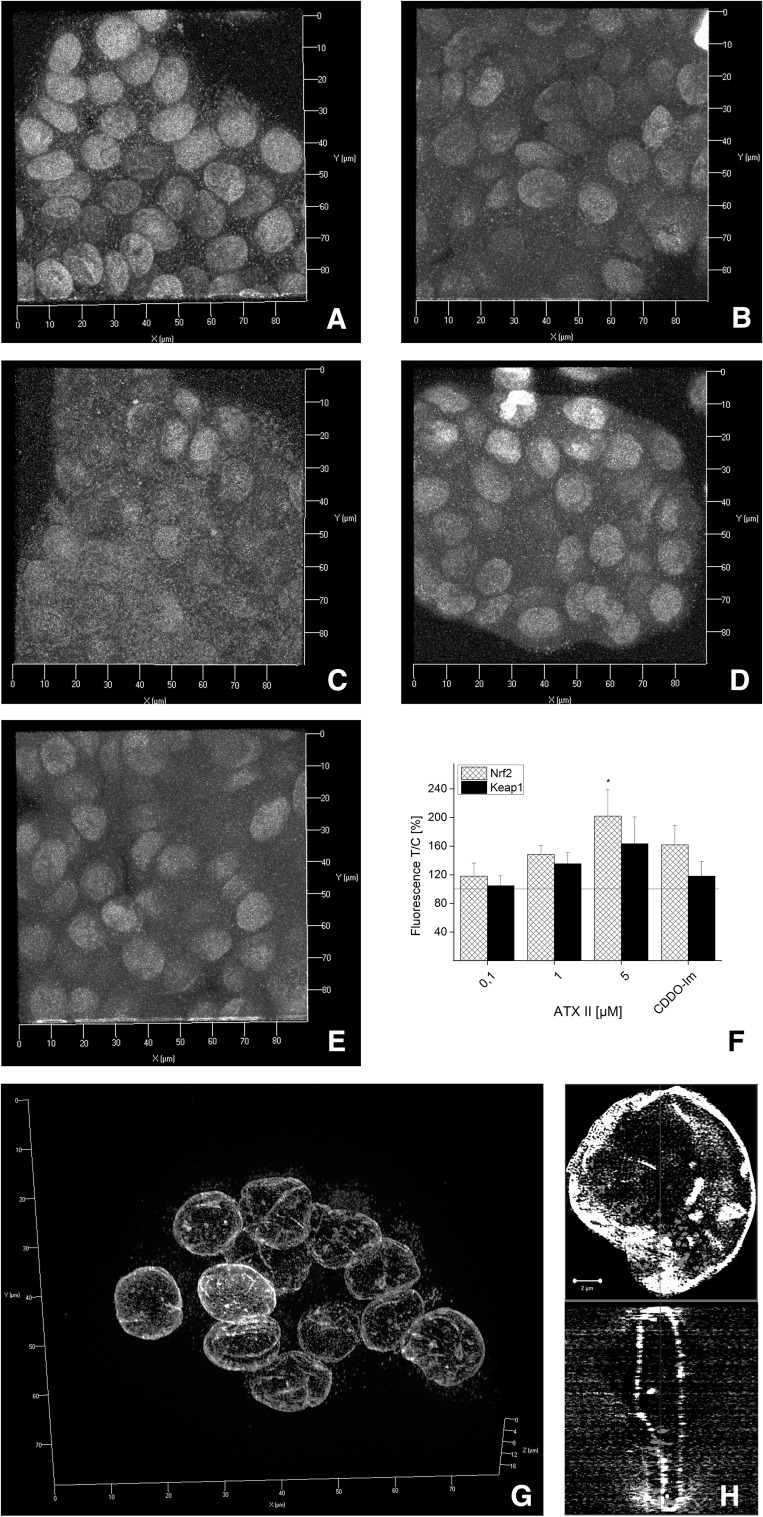
Immunofluorescence localization of Nrf2 and Keap1 after 1 h of incubation with ATX II in HT29 cells. Representative images of immunolocalization of Nrf2 (*red*), Keap-1 (*green*) and lamin B (*gray*) after incubation with EtOH (solvent control, **a**), 0.5 µM CDDO-Im (**b**), 0.1 µM ATX II (**c**), 1 µM ATX II (**d**), 5 µM ATX II (**e**). *Axes* *x* 0–90 µm, *y* 0–90 µm.   **f** Quantification of Nrf2 (*patterned bars*) and Keap1 (*black bars*) signals in cells incubated with ATX II (0.1, 1 and 5 µM) and CDDO-Im (0.5 µM). Mean values of fluorescence in the optical fields are expressed as percentage of solvent controls ±SEM (*n* = 3–6 independent experiments; significance levels indicated as *asterisk* refer the comparison with 0.1 µM ATX II, *p < 0.05). SIM 3D image of Nrf2 (*red*) and lamin B (*gray*) after incubation with 5 µM ATX II (**g**; *axes* *x* 0–80 µm, *y* 0–80 µm, *z* 0–20 µm) and representative cross section of one of the nuclei (**h**; *scale bar* 2 µm) (color figure online)

In order to further study the mechanisms sustaining the effects of ATXs on the Nrf2 pathway, additional immunofluorescence experiments were performed in HT29 cells. This model was previously used as representative of intestinal cells and it is particularly relevant for the study of food contaminants (Schwarz et al. 2012a; Tiessen et al. 2013b). In control conditions, the fluorescent signal of Nrf2 (red) appears to be similar to that of Keap1 (green; Fig. 3a). In cells incubated with CDDO-Im (1 h; positive controls), a tendency toward the increase in Nrf2 was visible, (Fig. 3b, f). After incubation with ATX I for 1 h (0.1, 1 and 5 μM; Fig. 3c–e), the signal of Nrf2 and Keap1 in HT29 cells was comparable to that of controls. In fact, quantification of the mean intensity of the fluorescence elicited by the antibodies recognizing Nrf2 and Keap1 presented minimal fluctuations. For instance, in cells incubated with 5 µM ATX I, the Nrf2 signal increase was only of 111 ± 4 % and for Keap1 of 117 ± 15 % (Fig. 3f). In contrast, 1 h of incubation with ATX II led to a concentration-dependent increase in Nrf2 fluorescence (Fig. 4c–f), which was significant at the highest tested concentration (202 ± 37 % increase for 5 µM ATX II; Fig. 4f). Evaluation of Keap1 signal in cells incubated with ATX II revealed only a tendency toward the increase, but no significant changes (163 ± 37 % increase for 5 µM ATX II; Fig. 4f). In order to better characterize the localization of Nrf2 after incubation with 5 μM ATX II, structured illumination microscopy (SIM) was also used. In fact, with this technique, it was possible to better visualize the presence of Nrf2 (red; Fig. 4g, h) inside the nuclear “shell” provided by lamin B (gray; Fig. 4g, h).

**Fig. 5:**
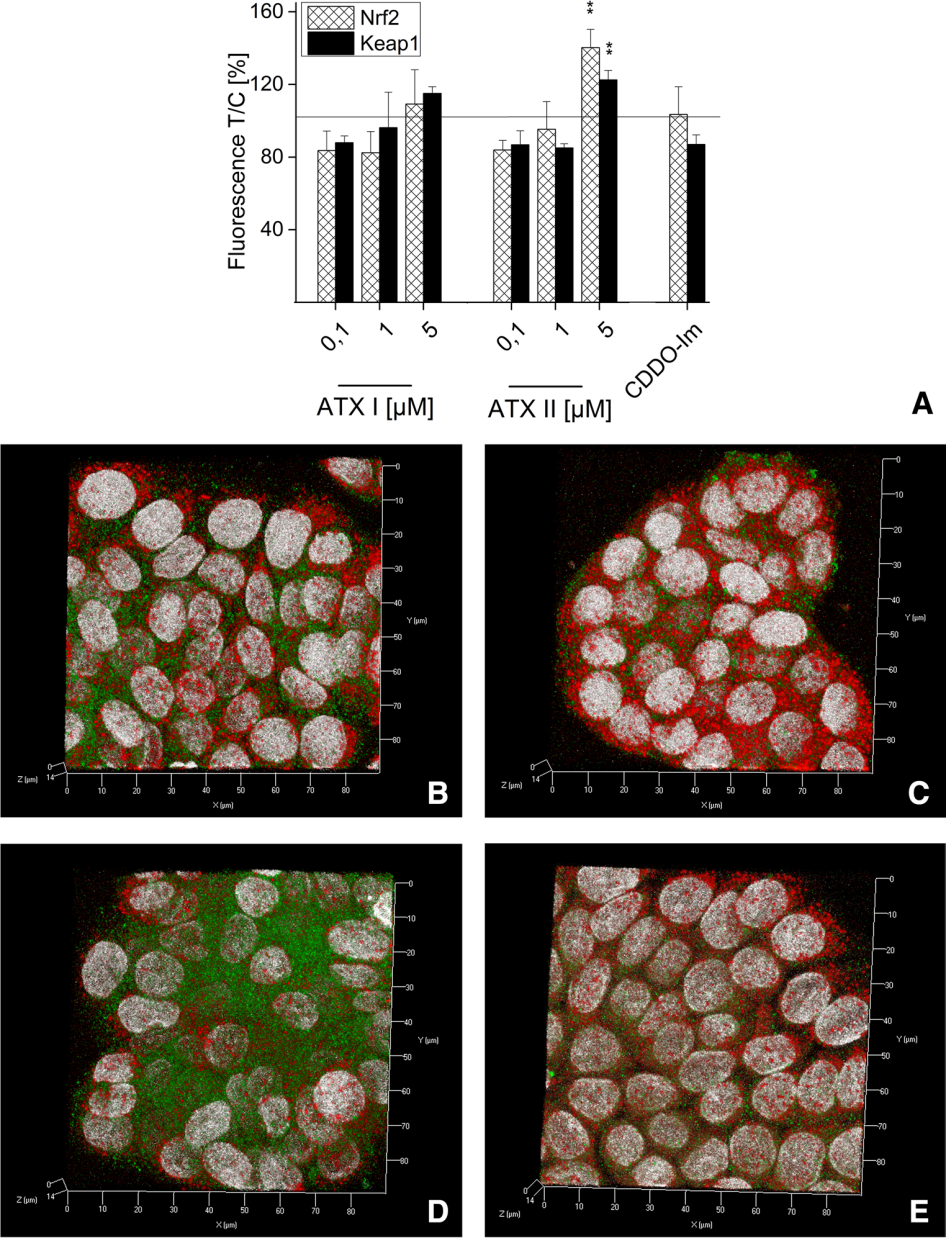
Immunofluorescence localization of Nrf2 and Keap1 after 3 h of incubation with ATX I and ATX II in HT29 cells. Quantification of Nrf2 (*patterned bars*) and Keap1 signals (*black bars*) in cells incubated with ATX I, ATX II and CDDO-Im (**a**). Representative images of immunolocalization of Nrf2 (*red*), Keap1 (*green*) and lamin B (*gray*) after incubation with 0.1 % EtOH (solvent control, **b**), 0.5 µM CDDO-Im (**c**) 5 µM ATX I (**d**), 5 µM ATX II (**e**). *Axes* *x* 0–90 µm, *y* 0–90 µm, *z* 0–14 µm. Mean values of fluorescence in the optical fields are expressed as percentage of solvent controls ±SEM (*n* = 3 independent experiments). Significance levels are calculated comparing signal intensity values normalized to solvent control (0.1 % EtOH) and *asterisk* refers to the comparison with 0.1 µM ATX II (**p < 0.01) (color figure online)

After 3 h of incubation, CDDO-Im did not seem to have an effect on Nrf2 and Keap1 in comparison with controls (Fig. 5a–c) and, in line with previous results, ATX I did not elicit any effect on the two proteins (Fig. 5a, d). Incubation of HT29 cells with ATX II for 3 h (Fig. 5a, e) triggered an increase in Nrf2 and Keap1 fluorescence, which was confirmed by image analysis with significantly higher signal intensities for both Nrf2 and Keap1 (5 µM ATX II; Fig. 5a, e).

**Fig. 6:**
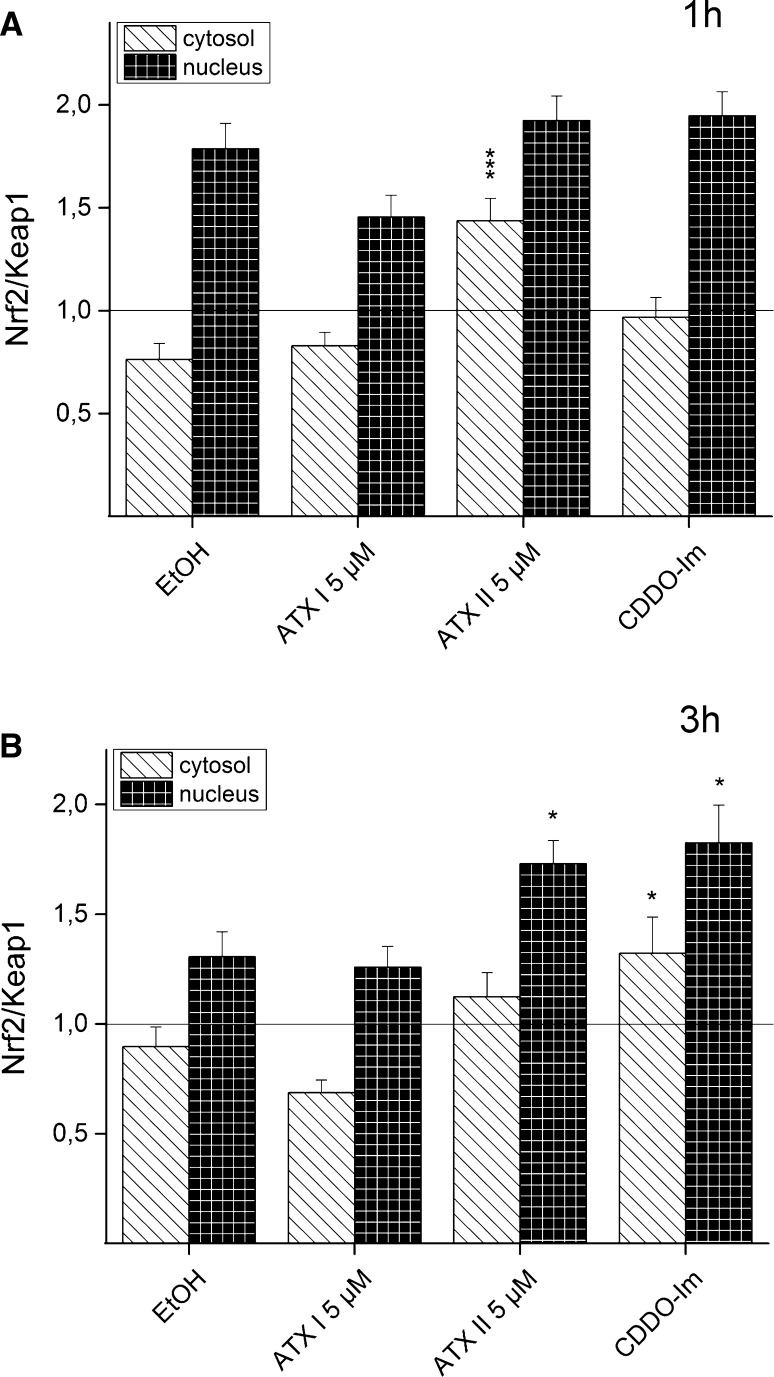
Influence of the incubation with ATX I and ATX II on the Nrf2/Keap1 ratio in HT29 cells. Nrf2/Keap1 fluorescence signal ratio were measured in the cytosolic (*striped bars*, *white background*) and nuclear (*patterned bars*, *black background*) compartments of HT29 cells. Results describe the incubation of HT29 cells with 0.1 % EtOH (solvent control), 5 µM ATX I, 5 µM ATX II and 0.5 µM CDDO-Im for 1 h (**a**) and 3 h (**b**). Mean values of fluorescence of Nrf2 and Keap1 for every compartment are expressed as ratio ±SEM. Significance levels indicated as *asterisk* refer the comparison with solvent control (EtOH; *p < 0.05, ***p < 0.001; *n* = 18 optical fields randomly selected from three independent experiments)

Moreover, in order to attempt to characterize the effect of our experiments on the relative distribution of Nrf2 and Keap1 in the nuclear and in the cytosolic compartments, an additional evaluation of the images was also performed. The mean intensity of fluorescence signals of Nrf2 and Keap1 in the selected areas (nuclei and cytosol) was used to express the corresponding Nrf2/Keap1 ratio signals. After 1 h of incubation with 5 µM ATX II, the cytosolic Nrf2/Keap1 ratio was significantly elevated in comparison with the solvent control EtOH, whereas no significant difference in the nuclear compartment was observed (Fig. 6a). After a longer incubation time (3 h), a significant increase in the cytosolic Nrf2/Keap1 ratio was observed for the positive control CDDO-Im. In addition, both CDDO-Im and 5 µM ATX II triggered in HT29 cells a significant increase in the Nrf2/Keap1 ratio in the nuclear compartment (Fig. 6b).

### Impact of ATXs on transcription levels of γGCL and Nrf2 in HT29 cells

**Fig. 7:**
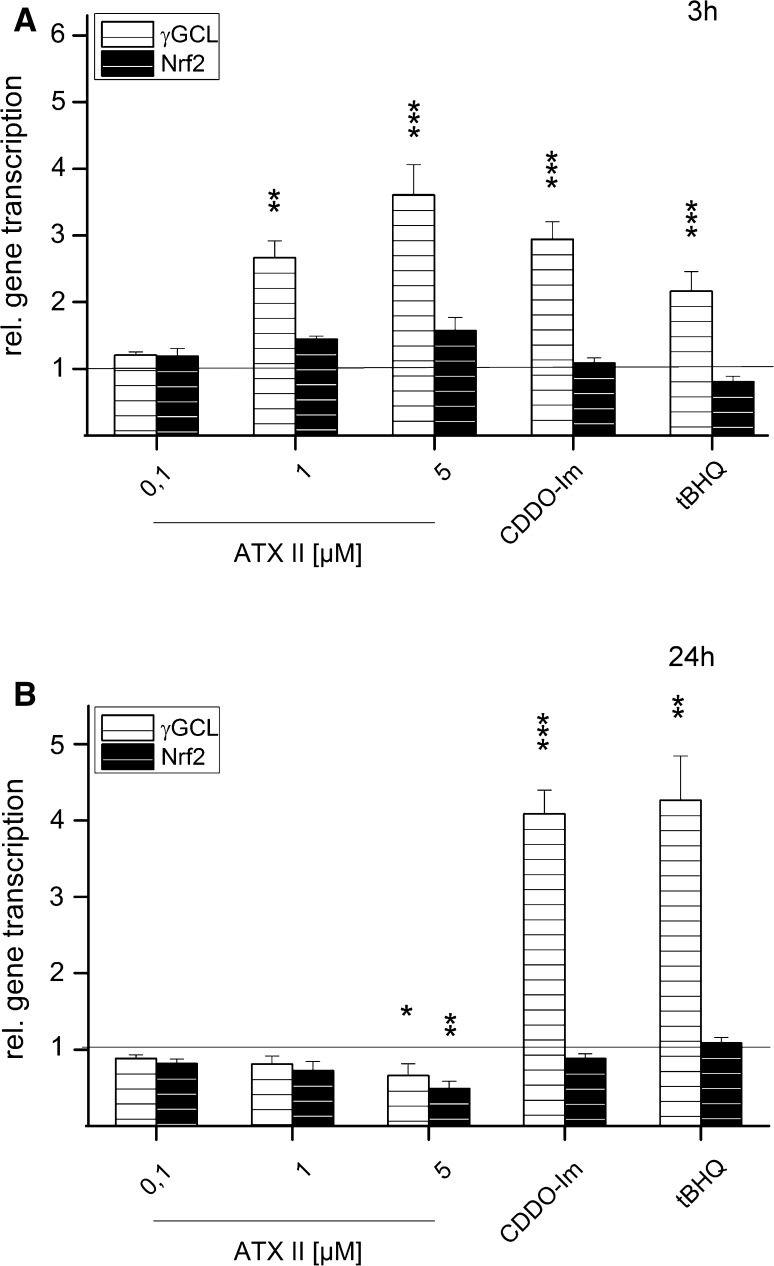
Concentration- and time-dependent effect of ATX II on gene expression of γGCL and Nrf2 in HT29 cells. Impact of ATX II on relative gene transcription of phase 2 enzyme γGCL (*white background*) and transcription factor Nrf2 (*black background*) in HT29 cells after 3 h (**a**) and 24 h (**b**). 0.1 % EtOH served as solvent control, 0.5 µM CDDO-Im and 200 µM tBHQ as positive controls. Depicted are mean $2^{{ -\Delta \varDelta C_{\text{T}} }}$ ±SEM as fold transcription of 3–5 independent experiments compared to solvent control. Significance levels indicated as *asterisk* refer to comparison with solvent control (EtOH; *p < 0.05, **p < 0.01, ***p < 0.001)

In order to verify whether in HT29 cells the effect of ATX II on Nrf2 activation and translocation could be further linked to an altered expression of ARE-dependent genes, the relative transcription of Nrf2 and γGCL was measured by qPCR. The gene transcription relative to the solvent control (0.1 % EtOH) of γGCL and Nrf2 was investigated after 3 h (Fig. 7a) and 24 h (Fig. 7b) of incubation with ATX II (0.1, 1 and 5 µM). 0.5 µM CDDO-Im and tBHQ (200 µM) were included as positive controls. After 3 h, a significant increase in γGCL mRNA was measured in cells incubated with CDDO-Im and tBHQ. Consistently, 1 and 5 µM ATX II also increased the relative gene transcription of γGCL of 2.7 ± 0.3 and 3.6 ± 0.5-folds, respectively. In cells incubated with ATX II, Nrf2 mRNA also showed a concentration-dependent tendency toward increase, even if not significant (Fig. 7a). After 24 h of incubation with ATX II, the trend was reversed, showing a significant decrease in both γGCL and Nrf2 at 5 µM (Fig. 7b).

### Impact of ATXs on mitochondrial activity in HT29 cells in the WST-1 assay

**Fig. 8:**
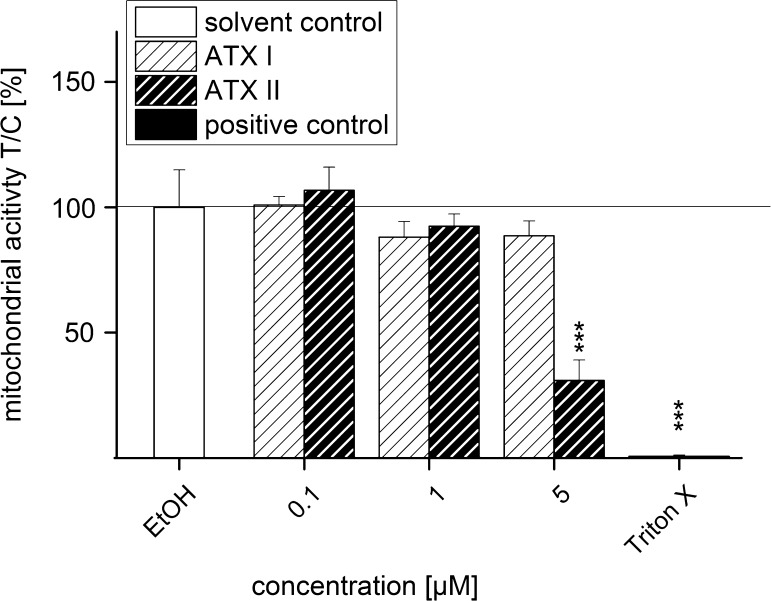
Cytotoxicity of ATX I and ATX II after 24 h of incubation in HT29 cells in the WST-1 assay. Mitochondrial activity of HT29 cells was measured after 24 h of incubation with ATX I (*striped bars*, *white background*) and ATX II (*striped bars*, *black background*) with the WST-1 assay. 0.1 % EtOH was used as solvent control and Triton X (0.1 %) as positive control. Data are expressed as mean values ±SEM of three independent experiments normalized to solvent control. Significance levels refer to a comparison to solvent control (EtOH; ***p < 0.001; *n* = 3 independent experiments performed in triplicate)

After 24 h of incubation with ATX II, the relative gene transcription of both Nrf2 and γGCL was decreased; since this behavior could potentially indicate cytotoxicity, the impact of the toxin on cellular viability was investigated. The cytotoxic potential of ATX II was determined with the colorimetric water-soluble tetrazolium salt-1 assay (WST-1) and ATX I was added for comparison. After 24 h incubation, ATX I did not reduce significantly the mitochondrial activity of the intestinal cells in any of the used concentrations (0.1, 1 and 5 µM), although a slight decrease for the two higher concentrations was observable (88 ± 6 % in case of 1 µM ATX I and 89 ± 6 % for 5 µM ATX I; Fig. 8). In contrast, ATX II showed a clear cytotoxic effect at the highest concentration tested (5 µM), reducing the mitochondrial activity significantly to 31 ± 8 %. From these data, an EC_50_-value for ATX II was calculated of 2.26 µM.

### Influence of ATX II on cellular tGSH and GSSG levels in HT29 cells

**Fig. 9:**
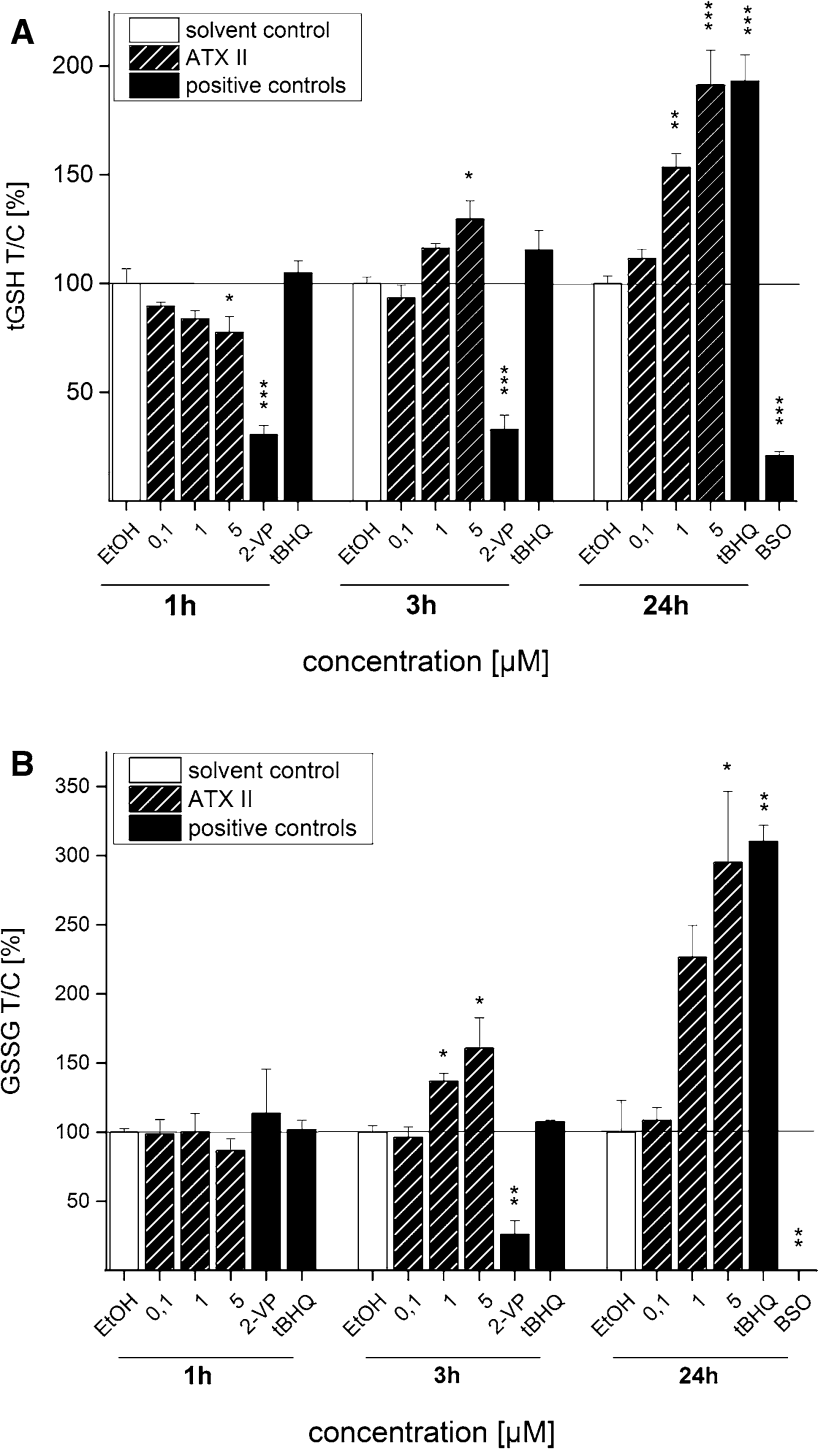
Concentration- and time-dependent effect of ATX II on tGSH and GSSG levels in HT29 cells. tGSH (**a**) and GSSG (**b**) content in HT29 cells after 1, 3 and 24 h of incubation with ATX II. 0.1 % EtOH was used as solvent control and 0.01 % 2-VP, 200 µM tBHQ and 1 mM BSO as positive controls. Depicted data are mean values ±SEM of 3–5 independent experiments normalized to solvent control. Significance levels indicated as *asterisk* refer to a comparison to solvent control (EtOH; *p < 0.05, **p < 0.01, ***p < 0.001)

In order to verify whether the effects observed on the gene transcription levels of γGCL induced by ATX II were mirrored in the actual levels of GSH, the concentrations of both tGSH (composed of GSH and GSSG) and GSSG were measured in HT29 cells after 1, 3 and 24 h of incubation with the mycotoxin (0.1, 1 and 5 µM). A concentration-dependent decrease in the tGSH was detected after 1 h of incubation with ATX II, and at 5 µM the tGSH levels were significantly lower in comparison with the solvent control (Fig. 9a). After 3 h, this effect was reversed, showing a significant increase in the cellular tGSH level at 5 µM ATX II (Fig. 9a). Incubation of HT29 cells for 24 h led to a further rise of tGSH, significantly elevated already at the concentration of 1 µM and above. In parallel with the response of tGSH, the levels of GSSG showed a tendency toward a slight decrease after 1 h of incubation with ATX II (5 µM) and increased significantly, in line with above-mentioned results, after 3 and 24 h (Fig. 9b).

### Effect of ATX II on reactive oxygen species formation in HT29 cells

**Fig. 10:**
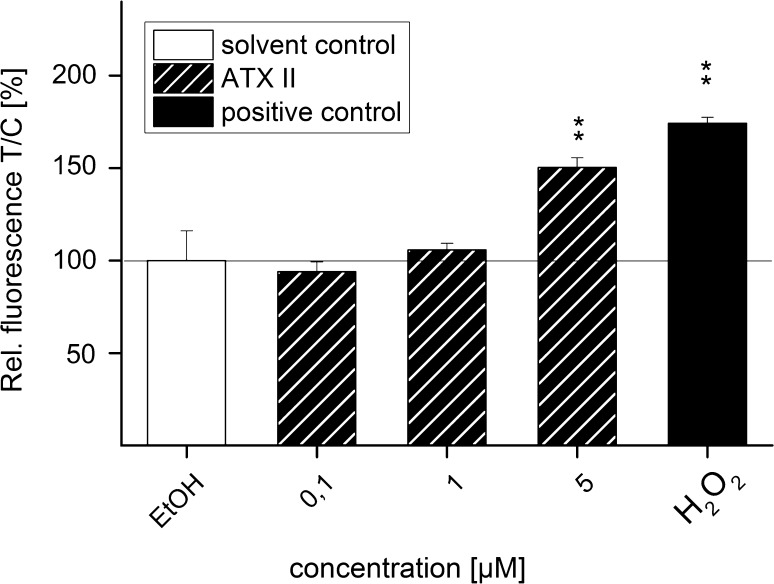
Oxidative potential of ATX II after 1 h of incubation in HT29 cells measured with the DCF assay. Fluorescence of DCF was measured after 1 h of incubation with ATX II in HT29 cells. 0.1 % EtOH was used as solvent control and 200 µM H_2_O_2_ as positive control. Depicted data are mean values ±SEM of three independent experiments performed in pentaplicates. Significance levels indicated as *asterisk* refer to a comparison to solvent control (EtOH; **p < 0.01)

In order to clarify whether the observed ATX II-mediated activation of the Nrf2-ARE pathway could be related also to oxidative stress, the potential of ATX II to induce ROS formation was investigated in HT29 cells using the dichlorofluorescein (DCF) assay. In this system, after 1 h of incubation, the fluorescence increased significantly (150 ± 5 %) for cells exposed to 5 µM ATX II (Fig. 10). The positive control 200 µM H_2_O_2_ led to a significant increase in signal intensity to 174 ± 3 % (Fig. 10).

## Discussion

The perylene quinone-type mycotoxins ATX I and ATX II, predominately produced by the saprophytic mold *Alternata alternata* (Lou et al. 2013), have been investigated for their toxicological importance with regard to their cytotoxic (Boutin et al. 1989; Fleck et al. 2012; Tiessen et al. 2013a), genotoxic (Fleck et al. 2012; Schwarz et al. 2012b) as well as mutagenic potential (Fleck et al. 2012; Stack and Prival 1986). However, studies focusing on the cellular metabolism and detoxification of these mycotoxins are relatively limited. In the present work, we investigated whether ATX I and ATX II interact with one of the major signaling pathways involved in phase II metabolism, the Nrf2-ARE pathway. The epoxide residue of ATX II, which is missing in ATX I (Fig. 1), confers sulfhydryl reactivity to the first, making ATX II a potential inducer of the Nrf2 transcription factor, which, in turn, is known to regulate the expression of a broad spectrum of phase II enzymes (Dinkova-Kostova et al. 2001; Itoh et al. 1997). In accordance with this mechanism, incubation with 5 µM ATX II for 20 h elicited a marked increase in Nrf2-ARE-dependent transcribed luciferase activity in the reporter gene assay, whereas incubation with ATX I had no effect on Nrf2 activation (Fig. 2).

Since the human exposure to mycotoxins is limited mainly to oral uptake, the subsequent investigations were conducted in the colon adenocarcinoma cell line HT29. In these cells, the effect of ATX I and ATX II on the transcription factor Nrf2 was studied with immunofluorescence and confocal microscopy after 1 and 3 h of incubation. These short incubation periods were chosen on the basis of previous studies that have demonstrated how Nrf2-ARE-dependent gene transcripts could be measured already after 3 h in this cell line (Boettler et al. 2011). For ATX I, no increase of in the Nrf2 signal was observed in any of our experimental conditions (Figs. 3, 5). On the contrary, a significant increase in Nrf2 immunolocalization was already observable after 1 h of incubation with ATX II (5 µM; Fig. 4) and was still detectable after 3 h (Fig. 5). To investigate the distribution of Nrf2 in the cellular compartments in more detail, its localization was also monitored with the help of SIM technique. As shown in Fig. 4, after 1 h of incubation with 5 µM ATX II, Nrf2 is clearly localized in the cellular cytoplasm, as well as, inside the nucleus (Fig. 4g, h). In addition to the effect on the Nrf2, ATX II also triggered an increase in the Keap1 signal (3 h incubation; Fig. 5). A similar effect was previously reported for the food contaminant deoxynivalenol (Katika et al. 2014), suggesting that Keap1 could also be an important target for the mechanism of action of mycotoxins. The synthetic triterpenoid CDDO-Im, even if it is a potent Nrf2-inducer in the luciferase assay (Fig. 2), did not trigger a significant increase in the Nrf2 signal in the microscopy experiments (Figs. 3, 4, 5). This difference can be probably explained by a distinct sensitivity and/or kinetics of Nrf2 activation triggered by CDDO-Im in comparison with ATX II in the two experimental systems. Moreover, previous studies already documented that the time dependency of CDDO-Im-mediated Nrf2 induction may vary between 0.5 up to 8 h of incubation in human leukemia cells (Liby 2005). As previously mentioned, Nrf2 is mainly located and repressed in the cytoplasm by Keap1 in its inactive state, so it was decided to present the immunolocalization data also as Nrf2/Keap1 signal ratios. Indeed, this more detailed analysis of the distribution of Nrf2 relative to Keap1 revealed a significant increase in the Nrf2/Keap1 ratios after 3 h of incubation with CDDO-Im in the cytosolic and the nuclear compartment in comparison with solvent controls (Fig. 6b). Interestingly, also ATX II led to a significantly elevated cytosolic Nrf2/Keap1 ratio already after 1 h of incubation (Fig. 6a), followed by a significant increase in the ratio in the nuclear compartment after 3 h (Fig. 6b). Intriguingly, this interpretation of the fluorescent signals could mirror a time-dependent increase in Nrf2 relative to Keap1 and its subsequent translocation from the cytoplasm into the nucleus.

As mentioned earlier, activation of Nrf2-ARE pathway triggers a signaling cascade which leads to the expression of, among others, genes that are associated with phase II metabolism. Consistently, 3 h of incubation with ATX II (1 and 5 µM) led to an increase in the gene transcription of phase II enzyme γGCL in HT29 cells, while no impact was observable on the amount of Nrf2 transcripts (Fig. 7a). The same pattern was observed for the positive controls CDDO-Im and tBHQ and was previously described also by Boettler et al. (2011). In particular, Boettler and co-workers described how chlorogenic acid is able to increase nuclear Nrf2 and the transcription of γGCL mRNA without directly altering the gene transcription of Nrf2. Overall, these data seem to suggest that the effect of ATX II and CDDO-Im in our experimental system could be sustained by a post-transcriptional mechanism, possibly targeting Nrf2 degradation rather than inducing a transcriptional change. This mechanism has been postulated in earlier publications as well, since it was described that Nrf2-inducers could act on the protein level, stabilizing Nrf2 and enhancing its activation, rather than interacting at the transcriptional level (Nguyen et al. 2003; Stewart 2002). After 24 h incubation with ATX II, the relative transcript levels of both γGCL and Nrf2 were comparable to that of the solvent control or even significantly lowered (5 µM ATX II Fig. 7b). To elucidate whether this reduction could be related to the onset of cytotoxic effects, the cytotoxicity of ATX II was investigated after 24 h with the WST-1 assay in HT29 cells. In line with previous findings, ATX II significantly reduced the activity of mitochondrial dehydrogenases with an EC_50_ of 2.26 µM (Fig. 8).

In order to give an overview of the ATX II-mediated Nrf2 activation from the nuclear translocation to the increase in the final enzymatic product, the impact of the mycotoxin on GSH and GSSG levels was also analyzed in HT29 cells. Incubation with ATX II for 3 h led to an increase in cellular tGSH (GSH + GSSG) and GSSG content (Fig. 9), which was in agreement with the time response of Nrf2 activation observed with confocal microscopy and qPCR. The ATX II-induced increase in tGSH and GSSG amount was still measurable after 24 h, whereas the gene transcription of γGCL was no longer elevated after that incubation time. On the contrary, after the short-term incubation of 1 h with ATX II, the cellular tGSH and GSSG levels decreased significantly. This reduction could indicate a potential consumption of GSH in the course of cellular defense mechanisms against toxic effects induced by ATX II, as, for instance, oxidative stress. In agreement with this hypothesis, the DCF assay indicated that ATX II triggers the formation of ROS in intestinal cells after 1 h of incubation (5 µM, Fig. 10). In case of ATX II-induced oxidative stress, the cellular depletion of GSH should go along with an increase in oxidized GSSG, which is then mirrored in a shift of the corresponding GSSG/GSH ratio (Nemeth and Boda 1989). Calculations of these respective GSSG/GSH values did not reveal any changes in cells incubated with ATX II, the ratios remained comparable to that of the solvent control. Based on this result, the consumption of GSH by ATX II-induced oxidative effects seemed less likely. Intriguingly, ATX II was previously reported to form adducts with GSH in a cell-free system (Fleck et al. 2014). Taking this observation into account, our data suggest that the prevailing effect of ATX II-induced reduction in GSH levels could be attributed to the formation of aforementioned GSH adducts, rather than pro-oxidative effects.

Overall, the present study demonstrates that the *A. alternata* mycotoxin ATX II may have an impact at several levels of the Nrf2 signaling pathway and that this impact is particularly relevant in intestinal cells. These findings open new insights into the comprehension of the toxicity of perylene quinone-type mycotoxins. In fact, even though it is commonly accepted that activation of the Nrf2 pathway is usually related to a reduction in the toxicity of the inducing compound, the interpretation of the presented data is probably more complex. Although induction of GSH is normally associated with a more efficient formation of detoxification products, this mechanism does not seem to play a major role for ATX II. In fact, previous studies demonstrated that a depletion of GSH in V79 cells prior to incubation with ATX II barely has any effect on the genotoxicity of the toxin (Fleck et al. 2014). In addition, activation of the Nrf2 pathway has also been described as a source of detrimental effects in cancer promotion (Singh et al. 2006). In this light, the possible involvement of Nrf2-ARE activation in carcinogenesis has also to be kept in mind when considering the genotoxic and mutagenic effects of ATX II (Schwarz et al. 2012b; Stack et al. 1986). Moreover, the observed Nrf2-ARE mediated induction of phase II response triggered by ATX II may, potentially, also enhance the health risk associated with the ingestion of food contaminated with other mycotoxins. In fact, since *Alternaria* spp. do not only produce altertoxins, but rather toxin mixtures, the phase I and II activating ability of ATX II may contribute to the intracellular formation of mycotoxin metabolites which may result in a further increase in overall toxicity of *Alternaria* fungi (Pahlke et al. 2016). In conclusion, the effects of ATX II on the Nrf2-ARE pathway seem to open new prospective in the study and the toxicological characterization of the emerging mycotoxins ATX I and ATX II.

## Materials and methods

### Fungi cultivation and toxin isolation

For the isolation of altertoxins (ATXs), rice was inoculated with the *A. alternata* strain DSM62010 and cultivated to obtain a toxin-containing ethyl acetate extract as previously described (Schwarz et al. 2012a). Briefly, ATX I was purified from the extract by the group of Thomas Hofmann, chair of Food Chemistry and Molecular Sensory Science at the University of Technology, Munich, Germany. The ethyl acetate extract was fractionated with solid-phase extraction; thus, extract aliquots (300 mg each) were dissolved in MeOH/H_2_O (90/10, v/v; 5 ml) and then eluted onto the top of a C18 ec cartridge (1 g, Chromabond, Macherey–Nagel, Düren, Germany) preconditioned with MeOH, followed by H_2_O. Fractionation was performed by flushing the column with decreasing polarity using different MeOH/H_2_O ratios: MeOH/H_2_O (30/70, v/v; 20 ml; fraction S1), MeOH/H_2_O (50/50, v/v; 20 mL; fraction S2), MeOH/H_2_O (70/30, v/v; 20 ml; fraction S3), MeOH/H_2_O (90/10, v/v; 20 mL; fraction S4), followed by MeOH/H_2_O (90/10, v/v; 20 ml; fraction S5), and methanol (40 ml; fraction S6). The fractions S1–S6 were collected and concentrated in a vacuum and freeze-dried. ATX I was isolated from fraction 4 by means of HPLC. Fraction S4 was dissolved in aqueous MeCN (1 ml, 50 %) and was chromatographically fractionated on a preparative HPLC system (PrepStar, Varian, Darmstadt, Germany) consisting of two HPLC-pumps (Model SD-1), a two wavelength UV detector (Prostar 325), fraction collector (Model 701) and equipped with a C-18 column (ThermoHypersil ODS, 10 × 250 mm, 5 μm; Kleinostheim, Germany) as the stationary phase. Monitoring the effluent (4.2 ml/min) at 350 nm, chromatography was performed starting with a mixture (58/42, v/v) of aqueous HCOOH (0.1 % in H_2_O) and MeCN, the MeCN content was increased to 50 % within 20 min, followed by column washing and reequilibration. Individual fractions were collected, concentrated under reduced pressure (40 °C) and freeze-dried in duplicate. ATX I was then repurified using the HPLC and column mentioned above and the following gradient. Monitoring the effluent (4.2 ml/min) at 350 nm, chromatography was performed starting with a mixture (80/20, v/v) of aqueous HCOOH (0.1 % in H2O) and MeCN; the MeCN content was increased to 45 % within 16 min and to 70 % within 4 min, followed by column washing and reequilibration. Individual fractions were collected, concentrated under reduced pressure (40 °C) and freeze-dried in duplicate, yielding ATX I (in high purity (>97 % HPLC–UV, 270 nm).

ATX II was isolated at the Department of Food Chemistry and Toxicology (University of Vienna) from the ethyl acetate extract obtained from *Alternaria*-infested rice with semi-preparative HPLC and verified by MS measurements on the ESI-Q-TOF MS (maXis classic, Bruker Corporation, MA, USA) as previously described (Schwarz et al. 2012b).

### Cell culture

Chinese hamster ovary cell line CHO-K1 cell line was purchased from ATCC (VA, USA), stably transfected with an expression vector for the *Photinus pyralis* luciferase driven by the ARE of murine GSTA1 gene (pGL4.22[luc2CP/Puro]) and an expression vector for enhanced green fluorescent protein (eGFP; pEGF-N1; ClonTech, CA, USA). CHO cells were cultivated in complete DMEM (10 % FCS, 1 % P/S) supplemented with 1 % l-glutamine (l-glu; 584 mg/ml) and 0.08 % puromycin (4 µg/ml). Human colon adenocarcinoma cell line HT29 was purchased from ATCC. HT29 cells were cultivated in DMEM supplemented with 10 % fetal calf serum (FCS) and 1 % penicillin/streptomycin (P/S, 50 U/ml), referred to as complete DMEM. Cell culture media and supplements were purchased from GIBCO Invitrogen (Karlsruhe, Germany), Lonza Group Ltd (Basel, Switzerland), Sigma-Aldrich Chemie GmbH (Munich, Germany) and Sarstedt AG&Co (Nuembrecht, Germany). Cell lines were cultivated and incubated in humidified incubators at 37 °C and 5 % CO_2_ and routinely tested for the absence of mycoplasma contamination.

### Nrf2 luciferase reporter gene assay

Nrf2 activation was measured in CHO cells carrying an ARE-dependent luciferase expressing plasmid and an expression vector for eGFP. 60 000 cells/100 µl complete medium supplemented with 1 % l-glu were seeded in 96-well plates and allowed to settle for 4 h. Cells were incubated with the respective concentrations of ATX I and ATX II with a final EtOH concentration of 0.1 % for 20 h. 0.1 % EtOH was used as solvent control and 0.1 µM 1[2-cyano-3,12-dioxooleana-1,9(11)-dien-28-oyl]imidazolide (CDDO-Im), a synthetic triterpenoid which activates the Nrf2-ARE pathway (Liby 2005), served as positive control. Afterward cells were washed with pre-warmed PBS and the plates were stored at −80 °C for at least 1 h. Subsequently 50 µl lysis buffer (no. E3791, Promega, Madison) was added and the cell lysates were transferred into a dark 96-well plate. Luciferase activity was measured with the TECAN GeniosPro plate reader. ATP (5 in 20 mM tricine) and luciferin (1 in 20 mM tricine) were auto-injected, and luminescence and eGFP-derived fluorescence signals were recorded for each well. Luminescence was normalized to the respective fluorescence of the eGFP value to account for eventual cytotoxicity. Each compound was tested in a triplicate with a minimum of three independent experiments.

### Immunocytochemistry and microscopy

For immunocytochemical analysis cells were seeded in multi-well microscopy slides (Falcon). After 72 h, cells were incubated with the respective toxin concentrations and CDDO-Im (0.5 µM) was used as positive control. At the end of the incubation (1 or 3 h), the cells were washed with pre-warmed PBS and fixed with 3.7 % formaldehyde for 15 min. The cells were then washed with PBS, and slides were stored at 4 °C prior to staining. Cells were permeabilized with 0.2 % triton X for 10 min, washed and blocked with PBS-BSA (1 %, 1 h, RT), the primary antibodies added and the slides incubated for 2 h (RT). Primary antibodies were purchased from Santa Cruz Biotechnology (Heidelberg, Germany) and used according to the specification of the supplier (1:1000 dilution): anti-lamin B goat polyclonal antibody (sc-6216), anti-Nrf2 rabbit polyclonal antibody (sc-722), anti-Keap1 mouse monoclonal antibody (sc-365626). After removal of the primary antibodies, fluorescent-labeled secondary antibodies were added and slides incubated in a dark humidified chamber for 1.5 h. For our study, Alexa Fluor 568 Donkey Anti-Rabbit (A10042), Alexa Fluor 647 Donkey Anti Goat (A-21447) and Alexa Fluor 488 Donkey Anti-Mouse (A-21202) antibodies were used (dilution 1:1500, Life Technologies). The slides were then washed and post-fixed with 3.7 % formaldehyde (10 min, RT); at the end of the post-fixation, 100 mM glycine was used to mask reactive sites and slides were mounted and sealed with Roti-Mount FluoCare (Roth, Graz, Austria). Images were acquired with a Confocal LSM Zeiss 710 equipped with ELYRA PS. 1 system. Structured illumination microscopy (SIM) images were acquired with Plan Apochromat 100×/1.46 oil objective, zoom 2, confocal images with a Plan Apochromat 63X/1.4 oil objective, zoom 1.5. Images were acquired from at least three different cell preparations for the analysis of Nrf2 and Keap1 signals and, for each cell preparation, at least three different optical fields were acquired, resulting in at least nine images for each experimental condition. Images were acquired to allow complete 3D reconstruction of the whole cells and Nrf2 and Keap1 quantification derived from the evaluation of the mean fluorescent signal intensity expressed as percentage of negative control (solvent, EtOH 0.1 %, Figs. 3, 4, 5). Mean intensity of the signals was derived from the raw data of the images with the software Zeiss ZEN 2012 SP1. The evaluation of the Nrf2 and Keap1 signals was performed in a central section of the 3D reconstruction, with the nuclear structure provided by lamin B as reference. During the selection of the optical fields and of the image analysis, the Nrf2 acquisition channel was initially disabled in order to avoid any bias. In order to ensure the comparability of the signals, the acquisition of all the experimental conditions (ATX I and ATX II 0.1, 1, 5 μM, positive and negative controls) of the same biological replicate was performed the same day, maintaining constant laser parameters (power, digital gain), as well as background levels. For the estimation of the nuclear and cytosolic ratio of the Nrf2/Keap1 signals (Mean intensity of signal of Nrf2 channel/Mean intensity of signal of Keap1 channel; Fig. 6), nuclei and cytosolic areas were randomly selected in the optical fields. The quantification was performed in at least two different areas of each compartment (nuclear or cytosolic) for every image, resulting in at least 18 different areas analyzed for each experimental condition.

### RNA isolation and measurement of transcription levels of γGCL and Nrf2 with qPCR

For the qPCR experiments, 800,000 (3 h incubation) and 500,000 (24 h incubation) HT29 cells were seeded in Petri dishes (Ø = 6 cm) and allowed to grow for 48 h. Subsequently, cells were incubated with 0.1, 1 and 5 µM ATX II and the solvent control EtOH (0.1 %) for 3 and 24 h in complete medium. CDDO-Im and pro-oxidant butylhydroquinone (tBHQ), which inhibits GSH synthesis and activates the Nrf2-ARE pathway (Huang et al. 2000), served as positive control. Subsequently, total RNA was isolated (RNeasy^®^ Mini Kit, Qiagen, Hilden, Germany) and reverse transcribed into cDNA (QuantiTect^®^ Reverse Transcription Kit, Qiagen, Hilden, Germany). Real-time PCR was performed with the StepOne Plus™ Instrument (Life Technologies) using QuantiTect SYBR Green PCR Master Mix and Primer Assays: HS_GCLC_1_SG, HS_NFE2L2_1_SG, HS_ACTB_1_SG (Qiagen Hilden, Germany). Transcript levels normalized to levels of the endogenous control gene β-actin were quantified using the ΔΔ*C*_T_ method as PCR efficiencies of target and control gene have been found comparable.

### WST-1 assay

Cytotoxic properties of ATX I and ATX II were investigated with the WST-1 assay (Roche, Basel, Switzerland). Briefly, 7 500 HT29 cells were seeded in 96-well plates and allowed to grow for 48 h at 37 °C and 5 % CO_2_ in a humidified incubator. Cells were incubated with 100 µl of complete medium, containing the respective toxin concentration in 0.1 % EtOH. The detergent Triton X (0.01 %) was used as positive control for cell permeabilization and subsequent cell death by necrosis (Borenfreund and Puerner 1985). Cells were washed after the incubation period of 24 h and WST-1 dye was added in serum-free medium. After 45 min the absorption at 450 nm was measured, values were normalized to respective control. The EC_50_ was calculated with OriginPro 9.1 with dose–response fitting.

### Measurement of cellular tGSH and GSSG

Concentrations of tGSH and GSSG in HT29 cells were measured according to Tietze (1969) and Schwarz et al. (2012b). Briefly, HT29 cells were seeded in Petri dishes (Ø = 10 cm) and allowed to grow for 48 or 72 h. Incubations took place for 1, 3 and 24 h with respective ATX II concentrations. 200 µM tBHQ was used as positive control for all three incubation times, 0.01 % 2-VP only for the 1 and 3 h periods due to its cytotoxicity, for the 24 h incubation, buthionine sulfoximine (BSO) was taken. For measurement of GSSG, 20 µl of 0.01 % 2-VP and 100 µl of 50 % triethanolamine (TEA) were added to 500 µl cell suspension and shaken for 1 h at 1300 rpm and 26 °C. 150 µl of cell suspension and 20 µl of GSSG standard solutions were mixed with 180 µl GSSG reaction mix composed of 154 µl A/B buffer [23.1 µl buffer A (125 mM KH_2_PO_4_, 8 mM EDTA) and 130.9 µl buffer B (125 mM K_2_HPO_4_, 8 mM EDTA)], 2 µl 6 mM 5,5′-dithiobis-(2-nitrobenzoic acid) (DTNB) in A/B buffer, 4 µl of 20 mM NADPH and 2 µl of GSH reductase (GR, 50 U/ml). GSH and GSSG samples were shaken for 30 s and then measured spectrophotometrically at 405 nm. The tGSH and GSSG values were referred to the respective protein contents, which were measured colorimetrically with the bicinchoninic acid (BCA) Protein Assay Kit purchased from Life Technologies (Thermo Scientific, MA, USA) and normalized to solvent control.

### Dichlorofluorescein assay

The influence of ATX II on the redox status of HT29 cells was measured with the DCF assay according to Keston and Brandt (1965). In this assay, 20,000 HT29 cells per well were seeded in 200 µl of serum-containing medium in a black 96-well plate and allowed to grow for 48 h. Afterward each well was washed with 37 °C PBS and incubated with 100 µl of dichlorofluorescein diacetate (DCF-DA) for 30 min at 37 °C. Subsequently, cells were washed with 37 °C PBS and incubated with the respective ATX II concentrations in a final EtOH concentration of 0.1 %, which also served as solvent control and 200 µM H_2_O_2_ was used as positive control. Phenol red-free DMEM was used for the incubations. The fluorescence was measured (excitation at 485 nm, emission at 528 nm) after 1 h and the values were normalized to solvent control.

### Statistical evaluation

All data are presented as the mean ± SEM. The data were tested for normal distribution with the Shapiro–Wilk test. The statistical significances for single concentrations (positive controls) were compared with the Student’s two-sample *t* test. Concentration ranges were evaluated for significant effects with the one-way analysis of variance (ANOVA), and in case of a significant difference of *p* < 0.05, post hoc Fisher’s least significant difference test was performed. Generally, raw data were used, but if the inter-experimental variances were too high, significances were calculated using the data normalized to controls. In this case, significances of samples were calculated compared to the lowest compound concentration used, where no effect was observed (0.1 µM ATX I or ATX II, respectively), since comparison to the 100 % controls was mathematically not feasible.

## Electronic supplementary material

Below is the link to the electronic supplementary material.

Supplementary material 1 (PDF 271 kb)
