# Supplementary material for: Activation of the Nrf2-ARE pathway by the Alternaria alternata mycotoxins altertoxin I and II
Source: Arch Toxicol. 2016 May 13;91(1):203–16. doi: 10.1007/s00204-016-1726-7 (PMC5225202; doi:10.1007/s00204-016-1726-7)
Supplement: Supplementary file 1 — Supplementary material 1 (PDF 271 kb) [file 204_2016_1726_MOESM1_ESM.pdf]

Supplementary Material

Activation of the Nrf2/ARE Pathway by the *Alternaria alternata* Mycotoxins Altertoxin I and II

Katharina Jarolim, Giorgia Del Favero, Gudrun Pahlke, Victoria Dostal, Kristin Zimmermann, Elke Heiss, Doris Ellmer, Timo D. Stark, Thomas Hofmann, Doris Marko*

* Corresponding author

Doris Marko

e-mail: doris.marko@univie.ac.at

Table S1

NMR data of ATX I

^{1}H NMR

H	Literature (300 MHz, CDCl3), Hradil et al. 1989	Literature ($CDCl_3$), Stack et al., 1986	NMR-measurement (500 MHz, CD_3OD)
1	7.83 d (J=8.8 Hz)	7.87 d (J=8.8 Hz)	7.99 d (J=8.8 Hz)
2	7.09 d (J=8.8 Hz)	7.01 d (J=8.8 Hz)	7.04 d (J=8.8 Hz)
3-O	-	12.7	-
5-α	3.02 m	ax. 3.17 m	3.15 m (J=3.0, 4.8, 14.4 Hz)
5-β	2.44 m	eq. 2.65 dt (J=15, 3 Hz)	2.64 m (J=8.0, 14.4 Hz)
6-α	3.18 m	ax. 2.43, dt (J=13, 3 Hz)	2.40 m (J=3.9, 5.5, 14.2 Hz)
6-β	2.70 m	eq. 3.17 m	3.08 m (J=2.8, 4.8, 14.2 Hz)
6a-O	-	-	-
6b	3.07 m	eq. 3.09 d (J=9 Hz)	3.03 d (J=8.8 Hz)
7	4.75 ddd (J=4.8, 9.1, 11.8 Hz)	4.78 ddd (J=12, 9, 5 Hz)	4.63 ddd (J=5.0, 8.9, 11.3, Hz)

7-O	-	-	-
8-α	3.08 m	ax. 2.94 dd (J=16, 12 Hz)	2.99 dd (J=15.8, 5.1 Hz)
8-β	2.92 dd (J=12.0, 16.1 Hz)	eq. 3.07 dd (J=16, 5 Hz)	2.91 dd (J=15.8, 11.4 Hz)
10-O	-	12.4	-
11	7.03 dd (J=0.9, 8.8 Hz)	6.92 d (J=8.8 Hz)	6.95 d (J=8.2 Hz)
12	7.83 d (J=8.8 Hz)	7.82 (J=8.8 Hz)	7.94 d (J=8.5 Hz)

^{13}C NMR data

**C**	**Literature CDCl$_3$), Stack et al., 1986**	**NMR-measurement (125 MHz, CD$_3$OD)**	**HMBC couplings**
1	132.7	133.8	-
2	119.5	119.6	-
3	162.3	163.1	1 and 2
3a	116.9	115.1	1, 2 and 5
4	205	207.0	5, 6 and 2
5	34.0	34.8	-
6	34.5	36.4	-
6a	69.2	70.0	1, 7, 6 and 5
6b	51.9	53.3	12, 7 and 8
7	66.1	66.7	8 and 6b
8	47.7	48.7	7 and 6b
9	202	204.9	11 and 8
9a	117.4	118.0	12, 11 and 8
9b	139.1	138.8	12, 11 and 6b
10	162.0	162.6	12, 11 and 6b
11	117.5	117.2	-
12	132.4	133.7	6b

12a	124.1	126.5	1 and 11
12b	122.7	125.3	6b, 12 and 2
12c	135.5	141.3	6, 2 and 1

^{1}H and ^{13}C NMR data of ATX I isolated from *Alternaria alternata* infested rice

Fig. S1

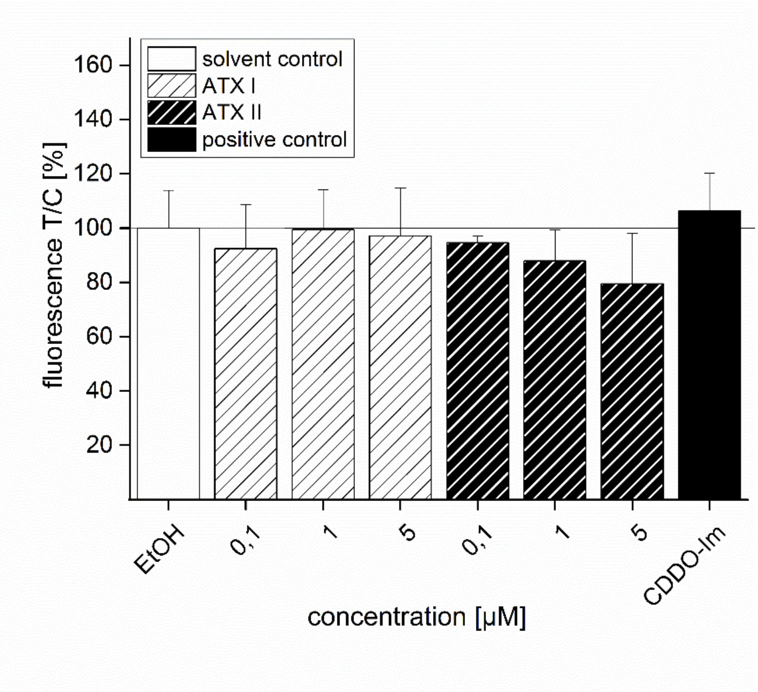

Fig. S1. Fluorescence of eGFP after 20h of incubation with ATX I and ATX II in CHO cells. Cells were incubated with ATX I (striped bars, white background), ATX II (striped bars, black background) and 0.1 µM CDDO-Im (positive control, black bars) in serum-containing medium. Relative fluorescence values are expressed as mean values ± SEM of at least 3 independent experiments, normalized to solvent control (EtOH).
